# Successful bilateral lung transplantation in pulmonary alveolar microlithiasis: A case report and review of literature

**DOI:** 10.1111/crj.13773

**Published:** 2024-05-09

**Authors:** Parviz Mardani, Reyhaneh Naseri, Reza Shahriarirad, Hadiseh Mahram, Masoud Shafi, Tahmoores Niknam, Mohammad Bagher Khosravi, Mohammad Javad Fallahi, Armin Amirian

**Affiliations:** ^1^ Thoracic and Vascular Surgery Research Center Shiraz University of Medical Science Shiraz Iran; ^2^ Shiraz Transplant Center, Abu‐Ali Sina Hospital Shiraz University of Medical Sciences Shiraz Iran; ^3^ School of Medicine Shiraz University of Medical Sciences Shiraz Iran; ^4^ Student Research Committee, School of Medicine Shiraz University of Medical Sciences Shiraz Iran; ^5^ Department of Cardiac surgery, Abu Ali Sina Hospital Shiraz University of Medical Sciences Shiraz Iran; ^6^ Anesthesiology and Critical Care Research Center Shiraz University of Medical Sciences Shiraz Iran; ^7^ Department of Internal Medicine Shiraz University of Medical Sciences Shiraz Iran

**Keywords:** cough, dyspnea, lung transplantation, pulmonary alveolar microlithiasis

## Abstract

**Background:**

Pulmonary alveolar microlithiasis (PAM) is a rare autosomal recessive genetic disorder with approximately 1000 known cases worldwide, in which calcium phosphate microliths deposit in the alveolar air spaces. As of writing this report, no definitive conventional therapy exists, and many PAM cases may progress to severe respiratory failure and potential death. Bilateral lung transplantation (BLx) seems to be the most optimal solution; however, this procedure is challenging along with limited reports regarding the outcome in PAM. We report a case of PAM successfully treated with BLx for the first time in Iran.

**Method:**

We present the case of a 42‐year‐old female with a longstanding history of cough, not responding to conventional antitussive medication, who was diagnosed as a case of PAM following a hospitalization due to coughing, dyspnea on exertion, and hemoptysis. Despite treatment with corticosteroid and medical treatment, no improvement was achieved and she subsequently developed respiratory and right ventricular failure, with oxygen ventilation dependence. Eventually, she was scheduled for BLx. The operation was successful and during her 2‐year follow‐up, no recurrence or significant postoperative complications has been reported.

**Conclusion:**

This case presentation and literature review confirm the effectiveness of BLx as a promising treatment for PAM‐diagnosed patients, improving both life expectancy and quality of life.

## Graphical abstract

Pulmonary alveolar microlithiasis (PAM) is a rare genetic disorder with no definitive therapy. Bilateral lung transplantation (BLx) offers hope for severe cases. We report the first successful BLx for PAM in Iran, supported by a review of similar cases worldwide. The procedure significantly improved the patient's condition during the 2‐year follow‐up, highlighting BLx as a promising treatment option for PAM patients with limited alternatives.

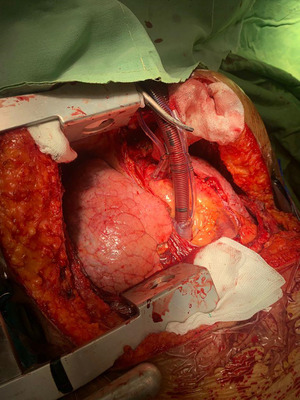

**Abbreviations**
BALF: bronchoalveolar lavage fluid
BLx: bilateral‐lung transplantation
CXR: chest x‐ray
ECMO: central extracorporeal membrane oxygenation
FEV1: forced expiratory volume in 1 s
FVC: forced vital capacity
HRCT: high‐resolution computed tomography
ICU: intensive care unit
PAM: pulmonary alveolar microlithiasis
PFT: pulmonary function test
TTE: transthoracic echocardiography
NA: not available

## 1 INTRODUCTION

Pulmonary alveolar microlithiasis (PAM) is a rare autosomal recessive disease in which a mutation in the SLC34A2 gene that encodes a sodium phosphate cotransporter is responsible for decreased phosphate clearance and widespread deposition of calcium phosphate crystals, called calcospherites or microliths, within the alveolar airspaces.^1^, ^2^ Currently, there is no established medical management to prevent the progression of PAM, and attempting to treat PAM empirically has been unsatisfactory; consequently, bilateral lung transplantation (BLx) remains the only definite viable treatment to change the course of the disease.^3^, ^4^ Of the more than 1000 cases reported worldwide,^5^ only 19 lung‐transplanted patients were reported in the literature.^6^ No recurrence has been stated after transplantation.^7^, ^8^ Herein, we described a case of severe PAM with a longstanding history, ultimately treated with a bilateral sequential lung transplant for the first time in Iran.

### 1.1 Case presentation

Our patient is a 42‐year‐old female without any significant past medical history who underwent BLx due to PAM. Her symptoms started 8 years ago when she visited a local hospital for a persistent productive cough. There was no history of smoking or possible occupational exposure. Her parents were nonconsanguineous, and there was no family history of pulmonary disease. At that time, she received nonspecific anticough therapy (dextromethorphan and pseudoephedrine) for symptomatic relief. However, her symptoms persisted, and despite episodes of relative relief and flare‐ups, she did not seek any medical attention.

**FIGURE 1:**
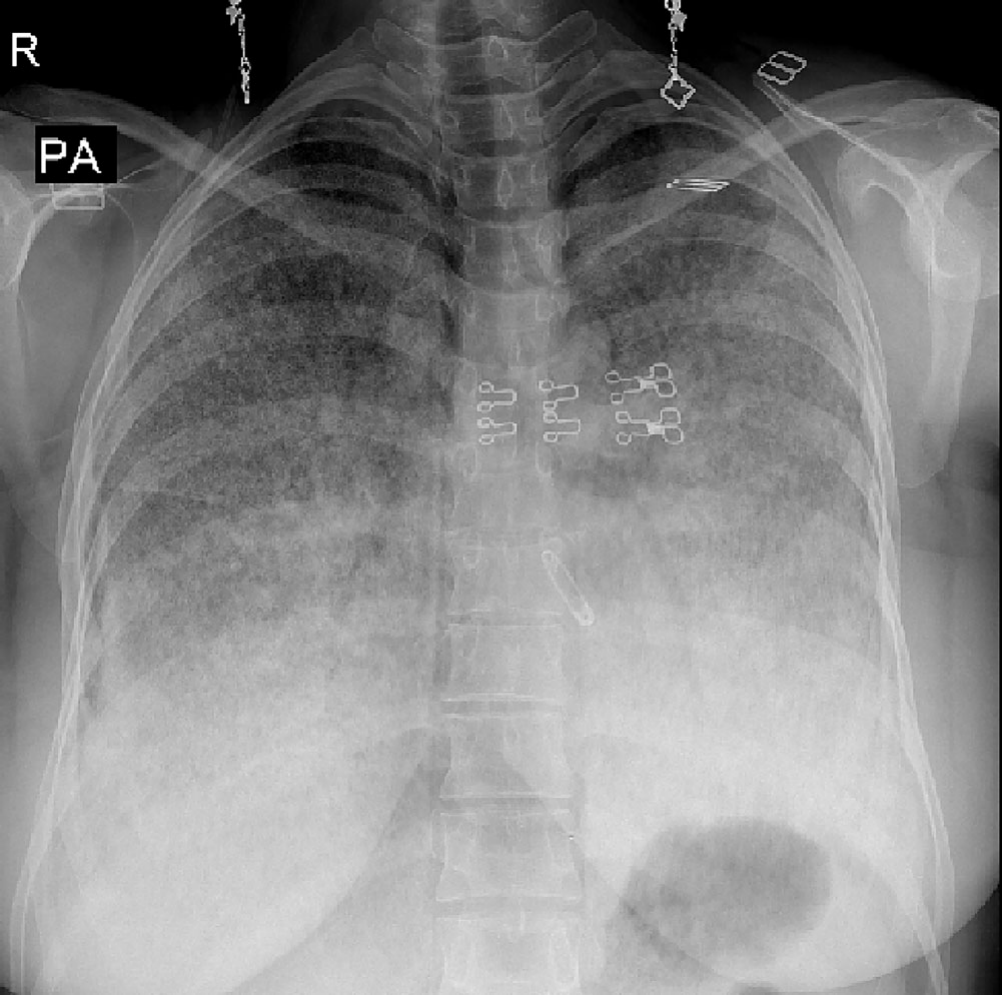
Chest X‐ray showing bilateral diffuse micronodular opacity involving all zones.

**FIGURE 2:**
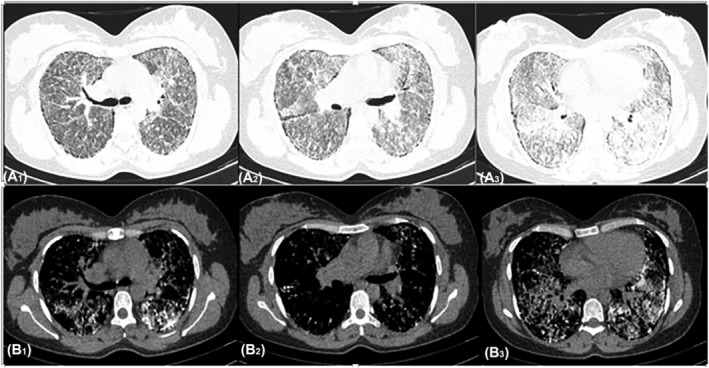
High‐resolution computed tomography of a patient with pulmonary alveolar microlithiasis. (A_1_, A_2_, A_3_) multiple bilateral calcified micronodules diffusely scattered over the lung parenchyma through three different sections in pulmonary window setting (B_1_, B_2_, B_3_) three different sections through the lung parenchyma showing combination of septal thickening and ground glass opacity illustrating “crazy paving sign” in the mediastinal window setting.

She was under conservative management for 2 years until she was hospitalized in our center because of chronic cough, excessive expectoration, progressive chest tightness, and dyspnea on exertion, accompanied by hemoptysis. Physical examination revealed clubbed fingers, bilateral inspiratory crackles in the middle and lower lung fields in chest auscultation, and oxygen saturation at rest of 93%. Routine laboratory findings (cell blood counts and serum chemistries) and inflammatory markers (body core temperature, leucocyte count, and C‐reactive protein) were unremarkable. A chest x‐ray (CXR) showed a diffuse bilateral micronodular pattern and a “sandstorm lung” appearance (Figure 1). High‐resolution computed tomography (HRCT) at the lung window demonstrated signs of apparent tiny cysts in subpleural spaces secondary to lung fibrosis, multiple calcified foci (up to 4 mm) in different parts of both lung fields, and a 10‐mm granuloma in the right middle lobe. It also showed diffuse interlobular and intralobular thickening associated with ground glass opacity, suggesting a “crazy paving sign” (Figure 2).

Moreover, arterial blood gas measurements and transthoracic echocardiography (TTE) showed no clinically relevant findings. On bronchoscopy evaluation, erythema of the respiratory mucosa was the only abnormality. Bronchoalveolar lavage fluid (BALF) did not reveal respiratory pathogens and was negative for malignant cells, tuberculosis bacterial cultures, and also polymerase chain reaction (PCR) was negative for respiratory viruses, *Pneumocystis jirovecii*, *Mycoplasma pneumonia*, and *Chlamydia pneumonia*. Moreover, BALF microscopic analysis unveiled intra‐alveolar lamellar microliths. Due to financial constraints, genomic analysis was not performed. However, we investigated her first and second‐degree relatives for any instances of lung disease or related symptoms, which was unremarkable. Thus, a positive diagnosis of PAM was established based on imaging studies and biopsy results. Following the diagnosis of PAM, she was discharged with a prescription of oral prednisolone (15 mg/day) and a salbutamol inhaler (two puffs every 6 h). The treatment was tapered gradually due to lack of efficacy.

**FIGURE 3:**
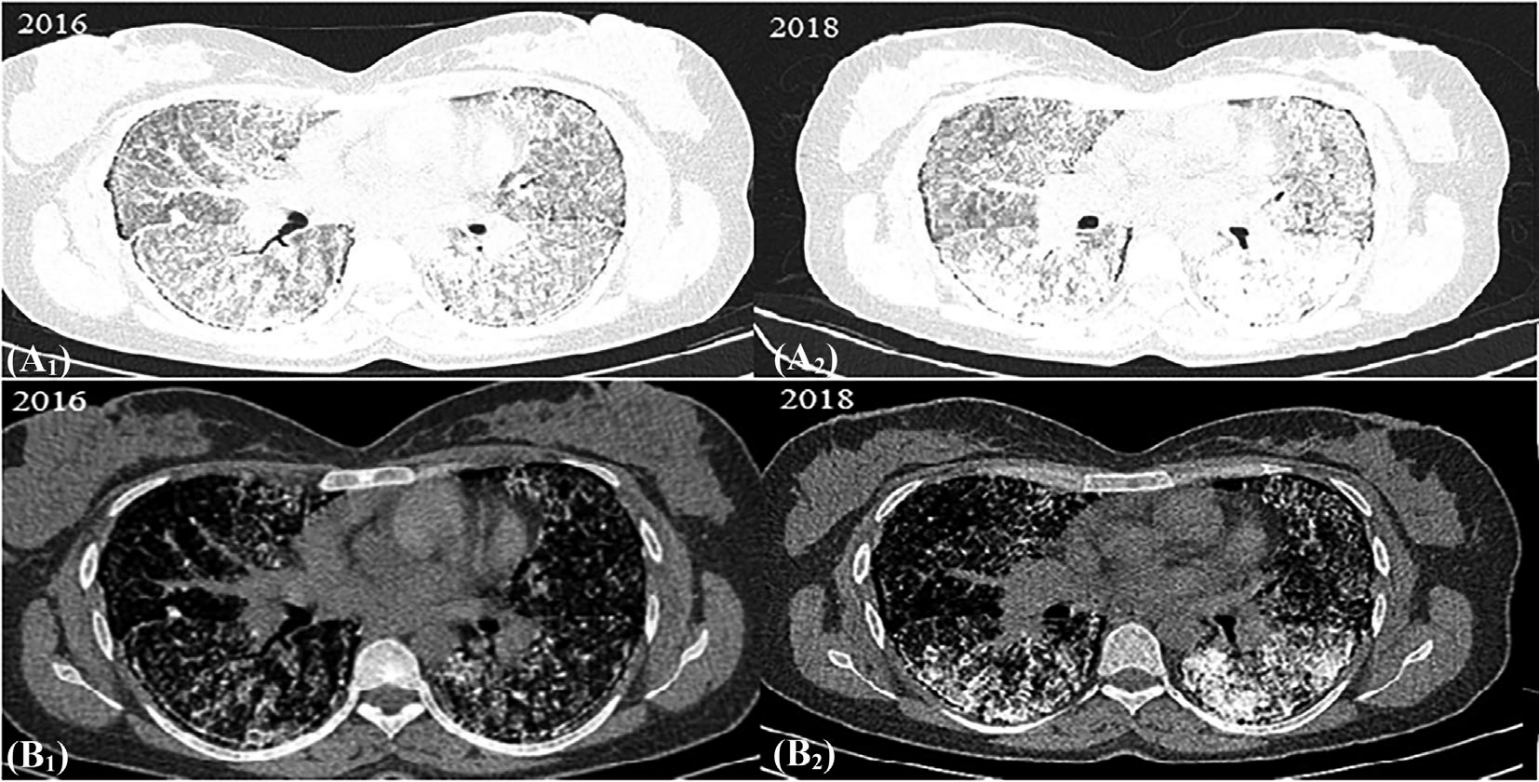
High‐resolution computed tomography of the patient with pulmonary alveolar microlithiasis over 2 years (A_1_, A_2_) a section of the lung in a pulmonary window setting, demonstrating expansion of fibrosis over the years (B_1_, B_2_) a mediastinal section of the lung, showing an increased number and size of calcified foci in B_2_.

Despite medication administration, she had progressively worsening respiratory distress and hypoxia and eventually required oxygen supplement (6–8 L/min) via nasal cannula. At the six‐year follow‐up, she was unable to perform the 6‐min walk test at baseline, with the peak of oxygen consumption within 5 m. TTE showed moderate pulmonary hypertension with pulmonary arterial systolic pressure of 50 mmHg in favor of cor pulmonale. A pulmonary function test (PFT) was suggestive of a restrictive ventilatory defect with a forced expiratory volume in 1 s (FEV1) of 1.5 L (53% of the predicted value), a forced vital capacity (FVC) of 1.55 L (47% of predicted) and an FEV1/FVC ratio of 0.96 (120% of predicted). Her second HRCT demonstrated an increased number and size of calcified foci in different parts of both lung fields and a greater extent of fibrosis compared with previous CT scans (Figure 3).

In view of worsening dyspnea and oxygen dependency, vast lung entanglement on CT, and decreased right ventricular function, she was hospitalized in our transplant center (Abu‐Ali Sina charity hospital, located in Shiraz, Iran) for BLx.

The patient was placed on lung transplant waiting lists pending availability of a donor, and pulmonary rehabilitation was included in the preoperative treatment plan. On November 2021, the patient underwent BLx with size and ABO‐matched donor after 5 months on the waiting list.

**FIGURE 4:**
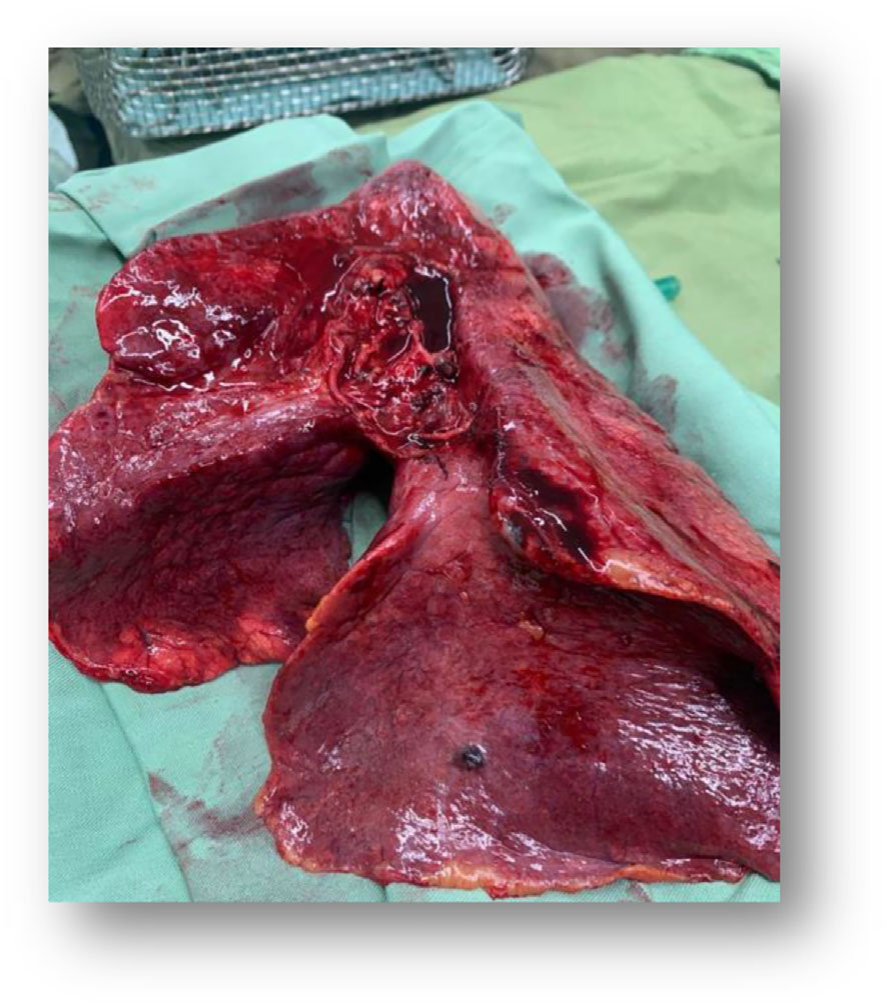
Gross view of the left lung of the donor for a lung transplant.

After general anesthesia, the procedure was performed by Clamshell incision. Once the chest was entered, the internal thoracotomy was completed posteriorly sparing the latissimus dorsi and serratus anterior muscles. The patient was connected to central extracorporeal membrane oxygenation (ECMO) after aortic and right atrium cannulas were inserted. After pneumonectomy, the implantation was conducted sequentially, starting with the most posterior anatomical structure, the bronchial anastomosis (Figure 4). At the end of the procedure, the patient was disconnected from ECMO and after the insertion of chest tubes, thoracotomy incision was closed. Total ECMO time was about six and a half hours without any major intraoperative complications. The initial medical therapy regimen consisted of methylprednisolone 1 g, tacrolimus 1 g, Cellcept 1 g, and broad‐spectrum antibiotics, including vancomycin 1 g, co‐trimoxazole 480 mg, piperacillin‐tazobactam 4.5 mg, and amphotericin 25 mg as per lung transplant protocol. Her vital signs were stable, and she was transferred to the intensive care unit (ICU).

**FIGURE 5:**
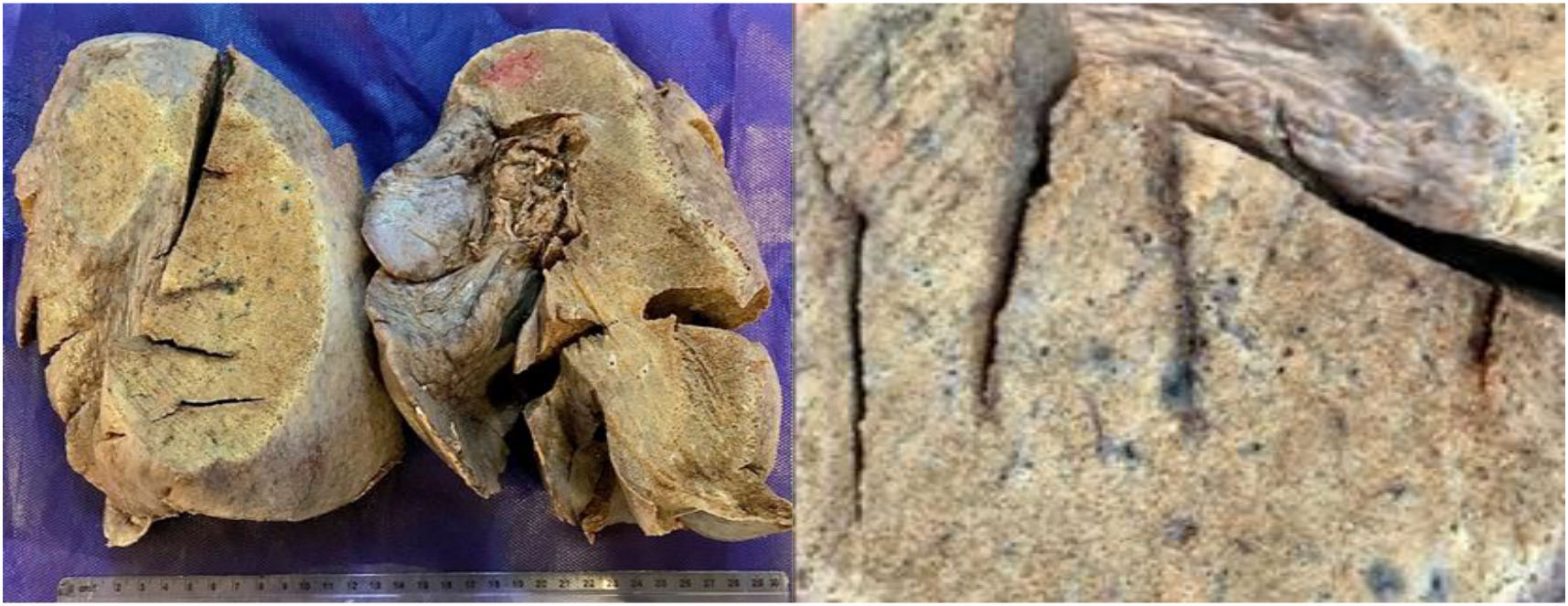
Pathologic findings in macroscopic view of the explanted lung in pulmonary alveolar microlithiasis. Cut sections of both lungs showing gritty‐granular surfaces.

**FIGURE 6:**
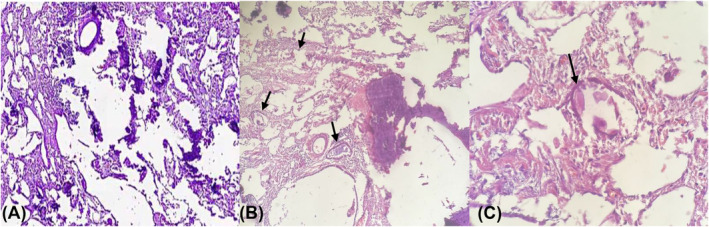
Gram‐stained histological section of lung tissue. Arrows show diffuse alveolar air space filled by lamellated calcifications and numerous introalveolar microliths. (A) and (B) magnification at 10×, and (C) magnification at 40×.

Cut sections of both lungs illustrated gritty‐granular surfaces (Figure 5), and histological examination showed characteristic features of PAM, including diffuse filling of alveolar air spaces by lamellated calcification (Figure 6).

**FIGURE 7:**
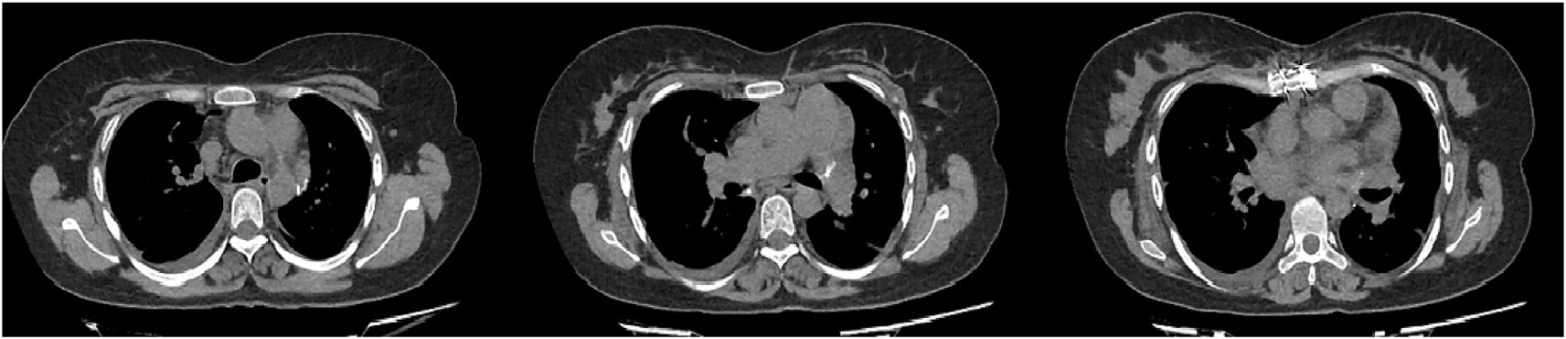
High‐resolution computed tomography of the patient with pulmonary alveolar microlithiasis, conducted at the first year postoperative follow‐up without any abnormality.

On the first postoperative day, she was successfully weaned off mechanical ventilation support, and nasal oxygen at a flow rate of 3 to 5 L per minute was provided. CXR was acceptable, and her TTE demonstrated acceptable right ventricle systolic pressure with SPAP of 30 mmHg. Immunosuppressive therapy was initiated with tacrolimus (2 mg twice a day), mycophenolate mofetil (1 g/day), and methylprednisolone (25 mg/day). On the fifth postoperative day, the patient presented with pneumonia symptoms, and she was treated with meropenem (1 g every 8 h) and levofloxacin (750 mg daily). She recovered uneventfully with complete remission. She was discharged from the hospital after 18 days of ICU admission with the continuation of the immunosuppressive regimen. In the first year of postoperative follow‐up, the patient had relatively acceptable quality of life without any symptoms regarding the procedure, and HRCT did not show any abnormality (Figure 7). During her second‐year follow‐up, the patient reported improved quality of life and no further complaints.

## 2 DISCUSSION

PAM is an autosomal recessively inherited pulmonary disorder with a limited number of cases, and no medical therapy except transplantation has been proven as a successful and reliable treatment method.^4^, ^5^ Herein, we presented the case of a 42‐year‐old female who went undiagnosed for 2 years despite experiencing symptoms of cough. Following confirmation of the diagnosis, she not only failed to show improvement with corticosteroid and medical treatment but also developed secondary pulmonary hypertension, progressive dyspnea, and hemoptysis. She ultimately underwent BLx and reported improved quality of life and respiratory symptoms during her 2‐year follow‐up. We believe that our report, among all others, could add to the body of evidence regarding the satisfactory treatment of this rare entity.

Our literature search revealed a total of 27 published cases with PAM who underwent lung transplant, which we have summarized the features of these patients in Table 1. The average age of patients diagnosed with PAM was 31.33 (SD = 5.10) years, and 51.85% were females, and that the diagnosis is usually made between the second and fourth decades of life.^5^, ^25^ Previous reports have stated that family history is present in 37% to 56% of the cases, with female predominance (53.3%); however, our review demonstrated that only three (11.11%) patients had reported positive family history, and two out of three were male.^26^, ^27^ Environmental factors, such as smoking and infection, play a role in accelerating the disease progression, despite the autosomal recessive inheritance pattern.^28^ Among these cases, four (14.81%) patients had a history of cigarette smoking. Despite our described case, her history was not significant for smoking, any occupational exposure, or any pulmonary disease in her family. The mean age at transplant for all cases was 47.26 (SD = 1.81) years, with 22 patients receiving bilateral transplant. In terms of outcome, nine patients did not exhibit any postoperative complications. Table 1 demonstrates the clinical and demographical features of lung‐transplanted PAM patients based on our review of published literature up to date.

**TABLE 1:** Clinical and demographical features of patients with pulmonary alveolar microlithiasis undergoing lung transplant based on a review of published literature.

N. Patient	Author	Age at diagnosis (Y/o)	Gender	Family history	Social factors	Duration of symptoms (years)	Diagnostic modality	Age at transplant (Y/o)	Type of transplant	Postoperation complication	Outcome/follow‐up duration (months)
1	Bonnette et al.^9^	N/A	F	N/A	N/A	N/A	N/A	46	Double	Bronchiolitis obliterans, infection and rejection	Expired/20 months
2	Stamatis et al.^10^	29	M	N/A	N/A	28	Radiologic findings	32	Double	Bleeding of the inferior left bronchial artery, temporary renal failure, systemic fungal infections, and cytomegalovirus pneumonitis	Alive/18 months
3	Raffa et al.^11^	44	F	N/A	N/A	3	Open‐lung biopsy	47	Single	Bronchial stricture	Alive/12 months
4	Edelman et al.^12^	11	F	N/A	Cigarette smoker 1.5 Pack/Day	N/A	Open‐lung biopsy	36	Double	N/A	Alive/32 months
5		41	M	Negative	Cigarette smoker 1.5 Pack/Day	15	Open‐lung biopsy	56	Double	Persistent blood loss, hemodynamic instability, and progressive hypoxemia	Expired/5 days
6	Jackson et al.^13^	N/A	F	N/A	N/A	N/A	Radiologic findings	35	Single	N/A	Alive/7.5 years
7	Coulibaly et al.^14^	11	F	N/A	N/A	6	Open‐lung biopsy	49	Double	Infection	Expired/3 months
8	Shadmehr et al.^15^	N/A	M	N/A	N/A	N/A	N/A	32	Single	Hemodynamic instability, reperfusion pulmonary edema	Expired/5 days
9	Samano et al.^3^	47	M	Negative	N/A	3	Open‐lung biopsy	48	Double	Pulmonary reperfusion syndrome, distributive shock and acute renal failure	Alive/12 months
10	Shigemura et al.^16^	47	F	N/A	N/A	N/A	Open‐lung biopsy	63	Double	N/A	Alive/24 months
11	Siddiqui et al.^17^	48	M	N/A	Cigarette smoker	N/A	Radiologic findings	48	Double	N/A	N/A
12	Borrelli et al.^18^	N/A	F	N/A	N/A	N/A	N/A	64	Single	N/A	Alive/5 years
13	Gucyetmez et al.^19^	10	F	Positive	N/A	N/A	N/A	53	Double	N/A	Alive/12 months
14	Klikovits et al.^20^	16	F	N/A	N/A	7	N/A	32	Double	Primary graft dysfunction and sepsis	Expired/11 days
15		2	F	N/A	N/A	6	N/A	52	Double	Reperfusion‐edema, atrial fibrillation	Alive/74 months
16		20	M	N/A	N/A	14	N/A	34	Double	N/A	Alive/67 months
17		10	F	N/A	N/A	6	N/A	52	Double	N/A	Alive/35 months
18		15	F	N/A	N/A	10	N/A	62	Double	Atrial fibrillation	Alive/29 months
19	Ren et al.^21^	52	M	Positive	N/A	Since childhood	Trans bronchial biopsy	53	Single	Bronchial anastomosis granulation with stenosis, bacterial infection, and pleural effusion	Alive/12 months
20	Alrossais et al.^6^	37	M	Negative	Cigarette smoker	29	Radiologic findings	45	Double	Mild reperfusion injury, primary graft dysfunction	Alive/43 months
21	Jindal et al.^22^	54	F	N/A	N/A	N/A	Radiologic findings	54	Double	N/A	Alive/12 months
22	Ahmed et al.^23^	N/A	M	N/A	N/A	N/A	N/A	52	Double	Primary graft dysfunction, pleural effusion	Alive/33 months
23		N/A	M	N/A	N/A	N/A	N/A	52	Double	Primary graft dysfunction	Alive/22 months
24		N/A	M	N/A	N/A	N/A	N/A	38	Double	Hemothorax	Alive/15 months
25		N/A	M	N/A	N/A	N/A	N/A	51	Double	Hemothorax	Alive/13 months
26	Helmink et al.^24^	42	M	Positive	N/A	N/A	Surgical lung biopsy during a video‐assisted thoracoscopic surgery	48	Double	Pleural effusions, sternal dehiscence, anastomotic necrosis, minimal acute rejection (grade 1A), pneumonia	N/A
27	Mardani P et al. (Our report) (2022)	36	F	Negative	Negative	8	Trans bronchial biopsy	42	Double	Pneumonia	Alive/2 years
Overall; N = 27		31.33 ± 5.10 years	Male: 13 (48.15%) Female: 14 (51.85%)			11.25 ± 2.56 years	Radiology: 5 (18.51%) Genetic (0) Open lung biopsy: 5 (18.51%) Trans bronchial biopsy: 2 (7.4%) Video assisted: 1 (3.7%) Not available data: 14 (51.85%)	47.26 ± 1.81 years	Single: 5 (18.51%) Double: 22 (81.48%)	No complication: 9 (33.33%) Deceased: 5 (18.51%) Infection: 6 (22.22%) Pulmonary reperfusion syndrome: 4 (14.81%) Primary graft dysfunction: 4(14.81%) Pleural effusion: 3(11.11%) Rejection: 2 (7.4%) Major bleeding: 2 (7.4%) Hemodynamic instability: 2 (7.4%) Hemothorax: 2(7.4%) Multi organ failure: 2 (7.4%) Atrial fibrillation: 2 (7.4%) Anastomosis stenosis: 1 (3.7%)	26.50 ± 4.76 months

PAM is a heterogeneous disease with an extent of clinical traits from asymptomatic to significant symptoms with right‐sided heart and respiratory failure.^29^ It has been demonstrated that while some patients have a very slow rate of disease progression, others progress rapidly.^5^ Generally, symptomatic patients present with dyspnea on exertion and cough, which can be accompanied by expectoration of microliths as primary complaints, and symptoms of cor pulmonale occur as a terminal manifestation similar to our case.^30^ Other symptoms, including chest pain, hemoptysis, asthenia, and pneumothorax, have also been reported.^31^ Our patient mainly experienced chronic cough, therefore she remained undiagnosed and subsequently untreated. Physical examination is often normal, but as the disease progresses, patients may exhibit signs of digital clubbing, peripheral or central cyanosis, and bilateral fine crackles on lung auscultation.^31^ Furthermore, when large volumes of the lung are filled with microliths, it can result in a restrictive ventilatory function impairment with diminished perfusion capacity, as was evident in the preoperative PFT of our case.^29^

A peculiar aspect of PAM is clinical and radiological dissociation; generally, asymptomatic patients present with a characteristic picture of infiltrations as fine sand‐like calcific micronodules, called “sandstorm lung” on chest radiographs.^32^ Herein, the typical “sandstorm lung” appearance was also shown on CXR. HRCT is the most useful diagnostic imaging for PAM, and its common findings include ground‐glass opacities, subpleural linear calcification, confluent and diffuse calcified nodules, and dense consolidations.^33^ Calcifications are also visible along the bronchovascular bundles and at the central region of the bronchovascular tree.^33^ As the opacifications expand, mediastinal outlines become concealed and with the involvement of pleural serosa, a “white out lung” appears.^1^ Moreover, the combination of ground‐glass attenuation and septal thickening with calcified nodules resulted in a “crazy paving” pattern that was noticeable in the presented study.^34^ Additionally, paraseptal and subpleural emphysema can appear as air cysts in the upper lobes, and small thin‐walled subpleural cysts may illustrate the “black pleural sign,” which was also evident in preoperative HRCT of our case.^1^, ^5^

Although a positive diagnosis of PAM can often be established based on radiographic studies, at least another clinical feature is required to make it definite.^25^ A lung biopsy (transbronchial, open, or video‐assisted thoracoscopic surgery) can confirm the diagnosis by identifying concentric intra‐alveolar microliths in doubtable cases.^35^ Five out of 27 (18.51%) lung‐transplanted patients were diagnosed via radiological modalities, five (18.51%) by open lung biopsy, two (7.4%) patients through transbronchial biopsy, one (3.7%) through video‐assisted modalities, and genetic analysis had no place as a routine diagnostic test. At the time of the study, genetic testing was not performed in our case due to a remarkably financial burden, which is in accordance with further studies not using genetic methods. We conducted a transbronchial biopsy with virtual bronchoscopy because open lung biopsy is not feasible in all cases and is invasive. Histopathological findings show arranged microliths in concentric lamellae around an amorphous nucleus and are distinguishable from metastatic or dystrophic pulmonary calcifications.^35^ Moreover, a BALF analysis can occasionally reveal mild lymphocytosis and microliths but is usually inconclusive.^35^ In our case, the combination of transbronchial biopsy results and radiological observations confirmed the diagnosis of PAM.

To the best of our knowledge, no authorized medical or genetic treatment is currently available to slow down the progression of PAM. Treatment with systemic corticosteroids, calcium‐binding agents, and bronchoalveolar whole‐lung lavage have been determined to be ineffective and are used as palliative measures.^3^, ^7^ In our case, prednisolone was administered prior to the diagnosis of PAM, and based on possible other differential diagnosis, however was discontinued following the ineffectiveness and ultimate pathological confirmation of PAM. While we acknowledge the theoretical possibility of prednisolone influencing the PAM course, we believe the low dose, short duration, and prompt withdrawal minimize the likelihood of significant harm.^36^ Although it is unclear how the efficacy of prednisolone administration was measured in a patient who was treated with prednisone prior to transplantation, but it was said to have experienced partial improvement.^3^ Moreover, treatment with corticosteroids has shown to suppress symptoms of cough, dyspnea, and chest tightness in some cases, and symptoms were recurred with the cessation of the treatment.^37^ Additionally, the potential benefits of addressing the initial differential diagnoses with prednisolone outweigh the minimal risk in this specific case.

Up to date, lung transplantation is proven to be the only beneficial treatment for end‐stage cases. There is no clear recommendation about when to refer patients with PAM for lung transplant, but it should be considered prior to the onset of severe right ventricular dysfunction.^5^ The majority of the lung‐transplanted patients were female, with the mean age of diagnosis and transplant of 22 and 47 years, respectively.^6^ Decisions regarding the time at which a successful transplantation should occur is when either right heart failure or severe respiratory failure with a requirement for oxygen is present, as was the case in our study.^3^

Recurrence after transplantation has not been observed to date during the posttransplant period.^6^ The most prolonged recorded survival for PAM after lung transplantation is 15 years.^13^ A short period of postoperative follow‐up is an unfortunate limitation of our study. In terms of outcome, only one patient has been reported with symptoms of infections, and in the postoperative deceased cases after BLx, prolonged ECMO time (6 h) in combination with extensive pleural involvement has been reported to be in charge of uncontrolled hemodynamic instability and multiorgan failure.^6^, ^16^ Although the optimal surgical approach ultimately depends on individual patient factors and surgeon expertise, however BLx is preferred to the unilateral because one lung replacement causes persistent shunting of blood through the native lung, subsequently filling of the alveolar spaces, and the consequent creation of large areas of intrapulmonary shunts.^38^ In our case, we recommended lung transplantation for the patient as a potential option for a full recovery. Improved pulmonary function, reduced oxygen requirements, and increased right ventricular ejection fraction after BLx in this study is indicative of its importance in such cases. However, further evaluation is needed to investigate patient survival and to explore risk of recurrence after lung transplantation.

## 3 CONCLUSION

PAM is still a challenging disease due to its global rarity. Our review summarizes by far the largest number of PAM patients undergoing lung transplant. We aimed to contribute to the current understanding of this condition. Conventional therapy does not always offer benefits, and because the progression is unavoidable and the majority of patients will eventually present with respiratory and right‐sided heart failure, prompt diagnosis and treatment are vital. Transplant is considerably the treatment of choice to improve both life expectancy and quality in patients with end‐stage disease. Surgeons should consider the importance of performing bilateral lung transplantation in such cases regardless of the recurrence risk.

## AUTHOR CONTRIBUTIONS

Parviz Mardani and Armin Amirian were the chief surgeons who performed the surgical procedures. Reyhaneh Naseri and Hadiseh Mahram collected the data and drafted the manuscript. Reyhaneh Naseri and Reza Shahriarirad revised the manuscript. Masoud Shafi, Tahmoores Niknam, and Mohammad Javad Fallahi clinically contributed to this study. All authors proofread the final version of the manuscript.

## CONFLICT OF INTEREST STATEMENT

The authors declare that they have no competing interests.

## ETHICS STATEMENT

All protocols were approved by the Ethics Committee of the Shiraz University of Medical Science. Also, the study was carried out in compliance in accordance with the relevant guidelines and regulations and the Declaration of Helsinki.

## PATIENT CONSENT

Written informed consent for publication of the patient's clinical details and pathologic images was obtained from the patient. A copy of the consent form is available for review by the editor of this journal.

## Data Availability

The data that support the findings of this study are available from the corresponding author upon reasonable request.
